# Visual Fields at Presentation and after Trans-sphenoidal Resection of Pituitary Adenomas

**Published:** 2011-07

**Authors:** Renu Dhasmana, Ramesh C Nagpal, Rahul Sharma, Krishan K Bansal, Harsh Bahadur

**Affiliations:** Himalayan Institute of Medical Sciences, Dehradun, India

**Keywords:** Pituitary Adenoma, Visual Field, Trans-sphenoidal Resection, Mean Deviation, Visual Field Index

## Abstract

**Purpose:**

To evaluate visual field changes in patients with pituitary adenomas following trans-sphenoidal surgery.

**Methods:**

Eighteen patients with pituitary adenomas underwent a complete ophthalmic assessment and visual field analysis using the Humphrey Field Analyzer 30-2 program before and after trans-sphenoidal surgical resection at the Himalayan Institute of Medical Sciences over a one year period. Visual acuity, duration of symptoms, optic nerve head changes, pattern of visual field defects, and variables such as mean deviation and visual field index were compared.

**Results:**

Thirty-six eyes of 18 patients including 10 male and 8 female subjects with mean age of 35.1±9.9 years and histologically proven pituitary adenoma were included. Mean visual acuity at presentation was 0.29 logMAR which improved to 0.21 logMAR postoperatively (P = 0.305). Of 36 eyes, 24 (66.7%) had visual field defects including temporal defects in 12 eyes (33.3%), non-specific defects in 10 eyes (27.8%), and peripheral field constriction in 2 eyes (5.6%). Mean deviation of visual fields at presentation was −14.28 dB which improved to −11.32 dB postoperatively. The visual field index improved from 63.5% to 75% postoperatively. Favorable visual field outcomes were correlated with shorter duration of symptoms and absence of optic nerve head changes at presentation.

**Conclusion:**

Visual field defects were present in two thirds of patients at presentation. An overall improvement in vision and visual fields was noted after surgical resection. An inverse correlation was found between the duration of symptoms and postoperative visual field recovery, signifying the importance of early surgical intervention.

## INTRODUCTION

Pituitary adenomas are common lesions comprising 10 to 15% of all primary brain tumors.^1^ Incidental pituitary tumors are found in approximately 15% of autopsies.^2^ The majority of these lesions are histologically benign. Clinically, they present as functioning or non-functioning pituitary adenomas. Despite ongoing advances in the medical and radiotherapeutic management of pituitary tumors, surgical resection remains the therapy of choice for the vast majority of these lesions.^3^ Surgical resection is indicated in cases with progressive visual field deterioration. Trans-sphenoidal surgery is performed when adequate resection is possible while sparing the normal gland.^4^

A spectrum of visual manifestations has been reported with these tumors, ranging from the absence of any visual symptoms to severe visual field defects and loss of vision. The prevalence of visual field defects in pituitary adenomas varies from 37 to 96% in different studies.^5^–^7^ The most common visual field defect is bitemporal hemianopia. However, other types of visual field deficits may also be observed.

The current study was undertaken to study visual outcomes of trans-sphenoidal resection in patients with pituitary adenomas at a single institute over a one-year period.

## METHODS

This prospective interventional study was conducted at the department of ophthalmology and neurosurgery of the Himalayan Institute of Medical Sciences, Swami Ram Nagar, Dehradun, from April 2009 to March 2010. The study was approved by the institutional review board/ethics committee. Patients with pituitary adenoma undergoing trans-sphenoidal surgical resection were included in the study, after providing written informed consent.

### Inclusion Criteria

Patients over 10 years of age diagnosed with pituitary adenomas on radiological imaging.

### Exclusion Criteria

1. Patients with ocular media opacities.
2. Patients with glaucoma, choroiditis, retinitis pigmentosa, optic neuritis, or any other ocular pathology affecting the visual field.
3. Patients physically and/or mentally unfit for detailed ocular examination.
4. Patients in whom visual field testing was not possible.

### Outcome Measures

Each patient underwent a complete ocular examination including color vision and visual field assessment, preoperatively and one week and 3 months postoperatively. A detailed history was obtained and general physical examination was conducted for all patients. Visual field assessment was performed using the central 30-2 program of the Humphrey automated static perimeter (720i, Carl Zeiss Meditec, Jena, Germany). Brain imaging studies including computed tomography (CT) or magnetic resonance imaging (MRI) were performed pre- and postoperatively in all patients.

### Visual Field Protocol

Standard achromatic perimetry using the central 30-2 program on the Humphrey Field Analyzer with Goldmann size III target was performed in all subjects pre- and postoperatively. Reliability criteria were fixation loss less than 20%, with false positive and false negative errors less than 33%.^8^

Changes in visual fields were analyzed separately for the right and left eyes in all 18 patients (36 eyes). Mean deviation (in dB) was used to compare overall visual field changes using paired t-test. Categorical variables (patient age and duration of visual symptoms) were analyzed as potential predictive factors for recovery of visual field defects by the Kruskal Wallis H test, using SPSS software version 10 (SPSS Inc., Chicago, IL, USA). Improvement in visual acuity was defined as a difference of at least 0.3 in logMAR notations.

## RESULTS

**Figure 1:**
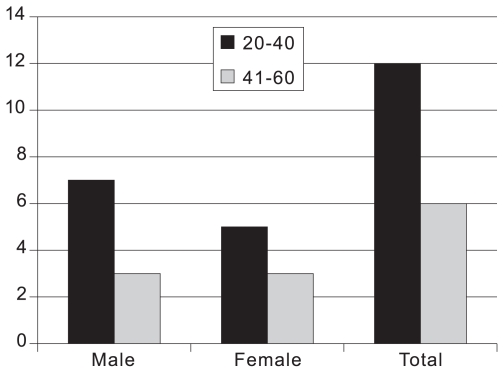
Age and sex distribution of the study population.

This series included 18 patients consisting of 10 male and 8 female subjects with mean age of 35.1±9.9 (range, 23 to 52) years and histologically proven pituitary adenomas. (Fig. 1). Visual acuity data are detailed in Table 1. Mean visual acuity was 0.29 logMAR at presentation and 0.21 logMAR postoperatively (P = 0.81, Table 2).

**Figure 2:**
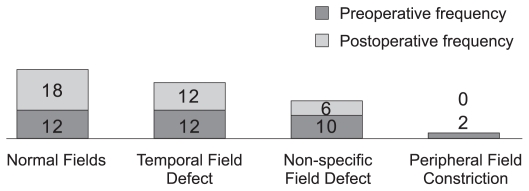
Pre- and postoperative patterns of visual field defects.

Out of 36 studied eyes, 24 (66.7%) had visual field defects at presentation, including 12 eyes (33.3%) with temporal defects, 10 eyes (27.8%) with non-specific defects, and 2 eyes with peripheral constriction (Fig. 2). The most common pattern of visual field loss was bitemporal defects, present in 6 (33.3%) patients.

Visual field data were recorded pre- and postoperatively. Average visual field index at presentation was 63.5% which was significantly improved to 75% postoperatively (P = 0.004, Table 2). Mean deviation at presentation was −14.28 dB which also significantly improved to −11.32 dB postoperatively (P < 0.005). Overall, visual fields improved in 17 (47.2%) eyes and remained unchanged in 19 (52.8%) eyes. Improvement in mean deviation was not correlated with age.

Preoperative examination revealed unilateral optic atrophy in 3 patients, bilateral disc pallor in 2 patients, and normal optic disc appearance in 13 patients. Significant visual field improvement was observed in the subgroup of patients with no evident optic nerve changes (P < 0.001).

Relative afferent pupillary defect was present in 4 eyes at presentation which did not improve in any of the cases. Defective color vision (discriminating less than 12 of 14 Ishihara color plates) was noted in 15 (41.6%) eyes at the time of presentation. Postoperatively, 12 eyes (33.3%) had defective color vision (P = 0.10). Two patients presented with unilateral third nerve palsy, one of whom had complete third nerve palsy.

Mean duration of symptoms was 5.8 months (range, 21 days to 1 year). The most common presentation was headache in 86.8% of patients. Five (13.9%) patients had visual symptoms. Duration of symptoms was less than 6 months in 7 (38.9%) patients. Postoperative improvement in visual field was greater in this subset of patients, as compared to subjects with longer duration of symptoms (P < 0.001, Table 3).

## DISCUSSION

Pituitary adenomas are generally slow-growing, benign neoplasms which can compress the anterior visual pathway, resulting in loss of vision. Anatomic relationships suggest that tumor extension 10 mm above the diaphragma sellae is necessary for the anterior visual pathway to become compressed. Trans-sphenoidal surgical resection or craniotomy can decompress the anterior visual pathway, leading to visual recovery. Visual improvement occurs in three phases, with the earliest phase of improvement taking place one week after surgery.^9^ It has been postulated that the initial improvement in vision is the result of recovery of nerve conduction. Later improvement is thought to be due to remyelination of decompressed optic pathways.^9^ Trans-sphenoidal surgery is the surgical treatment of choice for most pituitary adenomas because it is minimally invasive and highly successful.^7^,^10^

In the present study, visual acuity better than 6/12 was present in 28 (77.8%) eyes. Five eyes (13.8%) had visual acuity worse than 6/60. Other studies have reported visual acuity better than 6/12 in 65 to 72% of cases and less than 6/60 in 16.6 to 27.4% of eyes.^10^,^11^

At presentation, 12 (66.7%) patients had visual field changes while six did not. A range of visual field defects was noted; temporal defects were the most common abnormality, which is in accordance with existing literature.^12^ Non-specific visual field changes were present in 6 (27.8%) patients. Postoperatively, visual field defects improved and ultimately 18 eyes (50%) demonstrated normal fields. In other patients, the defects showed relative improvement after surgery. In another study, all cases had visual field defects with the most common being bitemporal defects noted in 50% of cases.^11^ Non-specific field changes were reported in 20% of patients in another study.^10^ Peripheral field constriction has been reported in a comparable percentage of subjects.^13^

Optic nerve changes are common in patients with pituitary adenomas. Longstanding compression by pituitary macroadenoma leads to optic atrophy. Five (27.8%) patients in our series showed optic nerve head changes; 3 patients had unilateral optic atrophy and 2 patients had bilateral disc pallor. A previously published series reported optic nerve changes in 72% of cases.^9^ Similar to our findings, the latter study demonstrated normal optic nerve appearance to be predictive of visual field improvement.

Automated static perimetry is the gold standard for visual field evaluation. In the current study, visual field recovery was examined with static perimetry as it can provide quantitative assessment of the visual field and incorporate tests for patient reliability while adjusting for field changes due to age, cataract, and small pupils; furthermore it is also less prone to examiner bias.^4^

We observed a statistically significant increase in both mean deviation and visual field index. Postoperatively, improvement in mean deviation occurred in 16 (44.4%) eyes. Another study reported improvement in mean deviation in 61% of operated eyes.^11^ The visual field index is a new center-weighted indicator on automated perimetry and has been suggested to reflect retinal ganglion cell function.^14^ The visual field index serves as an improved measure of visual field loss graded from 0 to 100%, where 100% is a perfect visual field. Currently, literature is available on the use of this index in analyzing glaucoma progression. To our knowledge, no study has evaluated the effect of pituitary adenomas on the visual field index. In the current study we observed a statistically significant increase in the visual field index postoperatively suggesting an improvement in retinal ganglion cell function. Visual field improvement was positively correlated with duration of symptoms less than six months; a finding which has been reported in another series.^10^

Ocular presentations other than visual symptoms have been described in the literature for pituitary adenomas. In the current study, two patients presented with unilateral third nerve palsy. There was no improvement after surgery in one patient with complete third nerve palsy, while the other demonstrated some improvement. The prevalence of ocular motor palsy in patients with pituitary adenomas ranges from 1 to 14%.^10^,^15^ Various mechanisms have been proposed to explain involvement of the third cranial nerve; these include pressure transmitted to the cavernous sinus by a growing or invading tumor, and compression of the third nerve between the tumor and the interclinoid ligament.^16^

Defective color vision has been reported in 56 to 71% of cases with sellar and suprasellar tumors.^17^,^18^ Mechanical compression of central or macular fibers by the tumor leads to loss of color vision.^17^ In the current study, color vision was affected in 41.6% of eyes which decreased to 33.3% of eyes postoperatively. All patients with color vision deficits had associated visual field defects. A recent study has suggested that recovery of visual field and visual acuity is more common than recovery of color vision.^19^ The reason for this phenomenon is unclear.

In summary, our series demonstrated that pituitary adenomas lead to visual field defects in 66.7% of patients. Trans-sphenoidal surgical resection resulted in an overall improvement in visual fields. Mean deviation and the visual field index also showed improvement after surgery. An inverse correlation was found between the duration of symptoms and postoperative visual field recovery, signifying the importance of early surgical intervention. There is a need for increasing awareness among the ophthalmic community and other physicians for timely referral of these patients and prompt neurosurgical intervention.

## Figures and Tables

**Table 1:** Distribution of eyes based on pre- and postoperative visual acuity

Snellen visual acuity	Number of eyes	

	Preoperative	Postoperative
>6/12	28	33
6/12 – 6/60	5	0
<6/60	3	3

**Table 2:** Pre- and postoperative visual function parameters

Visual parameters	Pre-operatively	Post-operatively
Mean Deviation (dB)	14.28	11.32
Visual Acuity (logMAR)	0.29	0.21
Visual Field Index	63.5%	75.0%

**Table 3:** Postoperative visual field changes categorized by duration of symptoms and optic nerve head changes

		Eyes	Number of Eyes	
			
			VF Improved	VF not improved
Duration	Less than 6 months	14	12	2
	More than 6 months	22	5	17

Optic nerve profile	With disc changes	7	1	6
	Without disc changes	29	16	13
